# GRAPES-DD: exploiting decision diagrams for index-driven search in biological graph databases

**DOI:** 10.1186/s12859-021-04129-0

**Published:** 2021-04-22

**Authors:** Nicola Licheri, Vincenzo Bonnici, Marco Beccuti, Rosalba Giugno

**Affiliations:** 1 University of Turin, Via Pessinetto 12, 10149 Turin, Italy; 2 University of Verona, Strada le Grazie 15, 37134 Verona, Italy

**Keywords:** Query processing, Pattern matching, Subgraph isomorphism, Graph indexing, Decision diagrams

## Abstract

**Background:**

Graphs are mathematical structures widely used for expressing relationships among elements when representing biomedical and biological information. On top of these representations, several analyses are performed. A common task is the search of one substructure within one graph, called target. The problem is referred to as one-to-one subgraph search, and it is known to be NP-complete. Heuristics and indexing techniques can be applied to facilitate the search. Indexing techniques are also exploited in the context of searching in a collection of target graphs, referred to as one-to-many subgraph problem. Filter-and-verification methods that use indexing approaches provide a fast pruning of target graphs or parts of them that do not contain the query. The expensive verification phase is then performed only on the subset of promising targets. Indexing strategies extract graph features at a sufficient granularity level for performing a powerful filtering step. Features are memorized in data structures allowing an efficient access. Indexing size, querying time and filtering power are key points for the development of efficient subgraph searching solutions.

**Results:**

An existing approach, GRAPES, has been shown to have good performance in terms of speed-up for both one-to-one and one-to-many cases. However, it suffers in the size of the built index. For this reason, we propose GRAPES-DD, a modified version of GRAPES in which the indexing structure has been replaced with a Decision Diagram. Decision Diagrams are a broad class of data structures widely used to encode and manipulate functions efficiently. Experiments on biomedical structures and synthetic graphs have confirmed our expectation showing that GRAPES-DD has substantially reduced the memory utilization compared to GRAPES without worsening the searching time.

**Conclusion:**

The use of Decision Diagrams for searching in biochemical and biological graphs is completely new and potentially promising thanks to their ability to encode compactly sets by exploiting their structure and regularity, and to manipulate entire sets of elements at once, instead of exploring each single element explicitly. Search strategies based on Decision Diagram makes the indexing for biochemical graphs, and not only, more affordable allowing us to potentially deal with huge and ever growing collections of biochemical and biological structures.

## Introduction

Graphs are a well-known mathematical structure used to encode relationships among elements of a set. They are employed in the representation of many biochemical systems at various levels from molecular representation [1], to protein-protein or RNA-mate interaction networks [2, 3] including also disease characterization [4]. The search for substructures, also called subgraphs, in biochemical systems is widely involved in many bioinformatic approaches as well as in the field of computational chemistry. Subgraph searching is a preliminary step in finding motifs in biological networks [5–7]. Network motifs are statistically over-represented sub-structures. They are building blocks of complex networks [8]. Several types of motifs have been discovered [9] such as the feed-forward loops that define patterns in gene regulatory networks [10]. Detection of motifs is a computationally challenging problem which requires the exhaustive search of subgraphs within a given network. Subgraph searching is also applied for tuning model parameters in biomolecular simulations [11]. In this context, graph-based representation of molecules facilitates the searching of fragments in large collections of molecules. Reliable model parameters are estimated based on the frequency of retrieved fragments. Moreover, collections of metabolic networks are queried in order to identify conserved pathways [12]. Because of the complexity of the querying task, many approaches limit the search to simple structures such as paths or small subgraphs [13]. Subgraph searching is also applied for biological network alignment, that is a powerful instrument for predicting functionalities of newly discovered elements [14]. Alignment can exploit the search of small subgraphs, also called seeds, within the set of networks that have to be aligned, in order to reduce the computational time requirements [15]. Other alignment tools, such as RINQ [16], use indexing schemes. Index-based strategy drives the alignment process to specific portion of the graphs and avoids expensive computations. Subgraph searching is also a baseline procedure in biomedical database systems^1^ consisting of genes, compounds, diseases, symptoms, side effects and annotations, integrated in networks. The networks are queried in order to prioritize gene-disease associations [17] or for drug re-purposing studies [18]. However, querying biological networks is a challenging task which, in many cases, increases its complexity with the query size [19].

The subgraph searching problem consists in finding a query graph within a target graph. It is a well-studied computational problem which is known to be NP-complete [20]. A generalization of such formulation considers more than one target graph. This is typically referred to as *one-to-many* in contrast to the original formulation that is referred to as *one-to-one*. Techniques for solving the one-to-one problem are mainly based on heuristics to speed-up the searching of a mapping function. Instead, the main efforts for solving the one-to-many problem are focused on developing a good filtering strategy for discarding target graphs belonging to the collection that do not contain the query graph. In particular, the most effective methodology for filtering strategy is the creation of an index in which features of the target graphs are stored. Then, when searching for a specific query graph, the target graphs are filtered by comparing the features of the query to those of the target graphs via the index. Thus, indexes are aimed at providing a very compact representation of the set of features and their correspondence to the target graphs. Performance in terms of construction time, size, querying time and filtering power are key concepts for their development. Such a performance is strictly related to the type of feature that is taken into account.

In details one-to-one approaches can be divided in two categories: *pure subgraph isomorphism* and *assisted solvers*. The first category is composed by algorithms that are focused on improving the performances of the combinatorial search by exploiting heuristic methods for pruning the search space, such as VF2 [21] and VF3 [22], or by changing the order in which query vertices are matched, such as RI [23]. The second category comprises algorithms able to efficiently reduce the number of target vertices that are candidate to match with query vertices. This reduction is obtained by indexing the target graph and by comparing the features assigned to target vertices with those of the query vertices. Indexing means that a predefined type of features are extracted from the graph and they are stored in a data structure in order to recognize in which parts of the graph, or in which graphs of a collection, a given feature occurs. Once candidates are retrieved, this information is also used for generating a quasi-optimal ordering of the query vertices. In this perspective, GraphQL [24] uses a pseudo subgraph isomorphism test, while TurboISO [25] exploits a tree-structured auxiliary index, and CSL [26] postpones Cartesian products with a matching order that prioritizes the query vertices in the core structure, similar to RI.

One-to-many approaches can be differentiated by the type of features they take into account (e.g. paths, trees, cycles or subgraphs) and how they extract them. GraphGrep [27], GraphGrepSX [28], GRAPES [29] and SING [30] extract paths by indexed graphs with simple enumeration procedures, but they differ in the type of data structure and additional information they use. Simple enumeration is also used by CT-Index [31] for extracting trees and cycles, and by GDIndex [32] and GCode [33] for extracting subgraphs. On the contrary, mining-based algorithms recognize *frequent* features with *ad hoc* procedures. SwiftIndex [34] and TreePi [35] extract frequent trees, as well as Tree+Delta [36] which also retrieves frequent substructures. Mining of subgraph is also performed by CP-index [37], gIndex [38], FG-Index [39] and Lindex+ [40]. Alternatively, signatures based on the pairs of vertex labels of the graphs can be exploited [41]. Mining-based approaches require high amount of time because of the mining step, however they are able to build more compact indexes with respect to the approaches based on the exhaustive enumeration.

In recent years, one-to-one approaches have reached a high performance. In many cases, they outperform the indexing methodologies of one-to-many approaches by simply scanning all the target graphs in a collection. However, when the number of graphs in the collection is relatively high, or when the target graphs have relatively large size, indexing techniques are still predominant, and hybrid approaches are investigated [42]. In [43], authors proposed an algorithm for the one-to-many problem which exploits a technique that it is usually embedded in one-to-one approaches, such as GraphQL, TurboIso and CFL. The technique consists in a pre-processing step for detecting the set of target vertices that are most probable to be matched with a given query vertices by looking at their connectivity. Authors have equipped the verification phase of GraphGrepSX, GRAPES and CT-Index with such a technique showing that modified one-to-many algorithm, in particular GRAPES, sensibly outperform GraphQL, TurboIso and CFL for the verification step. However, such a modification is added up to the original indexing techniques of the algorithms, thus it only helps in increasing the filtering power but it does not solve problems linked to the size and build time of the original index. Similar considerations can be done for cache-assisted frameworks [44, 45]. In this perspective, compression of the index plays a central role for both one-to-one and one-to-many approaches [46, 47].

A performance study [48] finds that index-based approaches have several issues in building indices on large graph databases in terms of number of distinct labels, number of vertices in data graphs, density of target graphs and number of target graphs due to their poor time and space efficiency of index construction. Among the tested algorithms, GRAPES showed the best performance in terms of running time. However, its index requires a relatively high memory amount compared to the other approaches. GRAPES is implemented both as sequential and parallel software using symmetric multiprocessing (SMP) architectures. In addition, GRAPES was developed for achieving good performance in collection of graphs as well as in scanning a query over a single large target graph. For these reasons, we decided to improve the performance of the sequential version of GRAPES by reducing the memory required for its index. We investigated the use of decision diagrams for reaching the goal without degrading the running time of the algorithm. Synthetic graphs were engaged for evaluating the performance of the modified version. In addition, a well established collection of biochemical graphs have been used for testing. Results show that the modified version, called GRAPES-DD, can reduce the size of the index of a factor of five orders of magnitude. The reduced index size helps the algorithm in optimizing accesses to primary memory, and as a result it can speed the building time of GRAPES-DD up with respect to the original algorithm in the same situations. GRAPES-DD is available at the following online repositories https://github.com/qBioTurin/grapes-dd and https://github.com/InfOmics/grapes-dd.

## Background

### Path-based graph indexing

Graph indexing strategies based on labelled paths consist in extracting all the paths in the graphs up to a given length (number of nodes which they are composed) and compactly storing them into a data structure [27, 28, 49, 50]. These techniques show good performance in terms of filtering power and construction/querying time. However, the size of the index is still a major problem with these approaches.

In what follows, we describe one of these techniques, called GRAPES [29]. GRAPES is the base methodology used here to apply MTMDDs (Multi-Terminal Multi-way Decision Diagram) to graph indexing.

#### GRAPES indexing

GRAPES [29] provides one of the most recent implementation of path-based graph indexing. It searches a query graph in one or more target graphs. For each path of the target graphs, GRAPES stores the identification of its starting vertices and the number of its occurrences in each graph. During searching phase, paths are extracted from the query graph and searched in the index. By comparing the ordered sequence of labels and the count of the occurrences, GRAPES effectively filters out target graphs which do not contain the query graph.

Formally, a path in a graph is an ordered sequence of vertices such that each vertex is connected with the next vertex by an edge. Thus, given a graph $G = (V,E)$, where $E:V\times V$, a path *p* of length *l* is a vector $(v^p_1, v^p_2, \dots , v^p_l)$ such that $v^p_i \in V$, for $1 \le i \le l$, and $(v^p_i,v^p_{i+1}) \in E$, for $1 \le i < l$. Given a set of labels $\Sigma$, a graph is labelled via a function $f_{\sigma }:\Sigma \mapsto V$ which maps each vertex to a label in $\Sigma$. The same label can be associated with different vertices. A labelled path ${\hat{p}}$ is obtained by mapping the vertices of a path to their corresponding labels via the $f_\sigma$ function, thus ${\hat{p}} = (f_\sigma (v^p_1),f_\sigma (v^p_2),\dots ,f_\sigma (v^p_l) ) = (\sigma ^p_1,\sigma ^p_2,\dots ,\sigma ^p_l)$. In GRAPES labelled paths are stored in a trie, a tree structure which compacts paths by their longest common prefixes. Given two labelled paths, ${\hat{p}}=(\sigma ^p_1,\sigma ^p_2,\dots ,\sigma ^p_l)$ and ${\hat{q}}=(\sigma ^q_1,\sigma ^q_2,\dots ,\sigma ^q_l)$, that share the first *i* labels, $(\sigma ^p_1,\sigma ^p_2,\dots ,\sigma ^p_i) = (\sigma ^q_1,\sigma ^q_2,\dots ,\sigma ^q_i)$, a branch, starting from the root of the tree, is built in order to represent the shared part of the paths. Then, the branch is split into two different branches that represent the non shared suffixes of the paths, $(\sigma ^p_{i+1},\dots ,\sigma ^p_l)$ and $(\sigma ^q_{i+1},\dots ,\sigma ^q_l)$. Information regarding the starting vertices, $v^p_1$ and $v^q_1$, is stored on the corresponding leaves of the branches, as well as the number of time each path occurs in each target graph. If only paths of the same length were extracted, the information would reside only on the leaves of the trie. By considering paths of variable length up to a maximum length $l_p$, the information also resides on intermediate nodes of the trie.

#### GRAPES filtering and verification

During querying phase, labelled paths are extracted from the query. Similarly to the extraction of paths from target graphs, for each path the number of times it occurs in the query graph is retrieved. Initially, all the target graphs are candidates to contain the query graph. Query paths are searched in the index in order to recognize the target graphs that contain the same paths of the query. For each path, the number of occurrences within the target graph must be equal or exceed the number of its occurrences in the query graph. By using the starting nodes of the paths stored in the index, the initial structures of target graphs are skimmed in order to extract only the vertices that are the starting point of paths in the query graph. Thus, the filtering procedure produces two different results, a list of graphs that may contain the query (since each selected graph contains the same labelled path of the query with the same amount), and for each selected graph the list of vertices that are candidate to match with the query vertices. The verification phase is performed with the VF2 algorithm [21] which solves the subgraph isomorphism problem. The problem of searching a query graph within a target graph consists in finding a mapping between the vertices of the query and target graphs such that constraints are satisfied. Constraints regard the compatibility of labels assigned to the vertices and the existence of the query edges between the corresponding query-target mapped vertices.

### Decision diagrams

Decision diagrams (DDs) are a broad class of data structures widely used to encode and manipulate functions efficiently [51]. Initially, they were proposed for industrial hardware verification due to their ability of encoding complex Boolean functions on very large domains. Then, they were successfully applied in different research fields ranging from the network reliability analysis [52] to the performance evaluation of stochastic systems [53]. In these contexts, DDs have proven to be an effective tool (1) to encode compactly structured sets by exploiting their structure and regularity; (2) to manipulate entire sets of elements at once, instead of exploring each single element explicitly.

In this paper we will focus on a specific type of decision diagram, which is called Multi-Terminal Multi-way Decision Diagram (MTMDD). Formally, an MTMDD is a rooted, directed, acyclic graph representing a function $f: \mathrm{I\!N}_{x_1} \times \dots \times \mathrm{I\!N}_{x_K} \rightarrow {\mathcal {R}}$ over a set of variables $\{x_1, \dots , x_K\}$, where $\mathrm{I\!N}_{x_k} \subset {\mathbb {N}}$ is the finite set of values that variable $x_k$ can assume, and ${\mathcal {R}} \subset {\mathbb {N}}$ is the finite set of possible function values [54]. An MTMDD node can be either *terminal* or *non-terminal*. A terminal node has no outgoing edges and is labeled with a constant $n \in {\mathcal {R}}$. A non-terminal node *m* is labeled with a variable $var(m) \in \{x_1, .. x_K\}$ and has exactly $N_{var(m)} = |\mathrm{I\!N}_{var(m)}|$ outgoing edges pointing to its children nodes. We refer to the *i*-th child of node *m* as *child*(*m*, *i*), with $0 \le i < N_{var(m)}$. The evaluation of the function represented by a given MTMDD, for a given assignment of its variables, can be determined by tracing a path from the root to one of the terminal nodes.

**Fig. 1:**
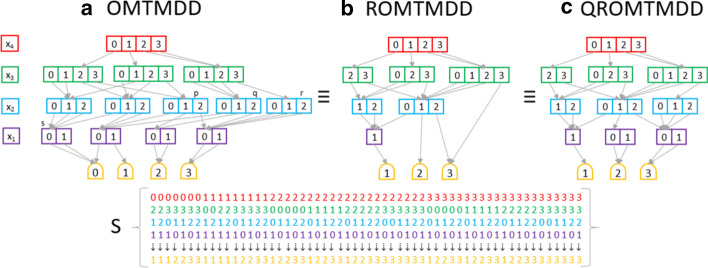
Different kind of MTMDD encoding the function counting the occurrences of an element into the multiset *S*: **a** an OMTMDD; **b** a ROMTMDD; **c** a QROMTMDD

**Fig. 2:**
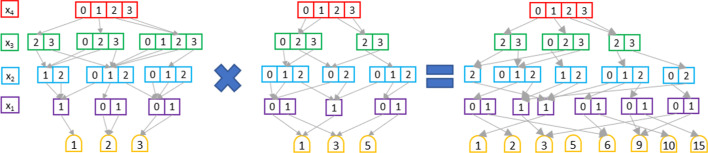
A multiplication operation between two QROMTMDD

A simple example of MTMDD is reported in Fig. 1a. This MTMDD encodes the function counting the occurrences of an element into a multiset *S*^2^ where each element is described by a tuple $(x_1, x_2, x_3, x_4)$ with $x_1 \in \{0,1\}$, $x_2 \in \{0,1,2\}$, $x_3 \in \{0,1,2,3\}$ and $x_4 \in \{0,1,2,3\}$. Thus, the MTMDD path from the root assuming $x_4 = 2, x_3 = 3, x_2 = 0, x_1 = 1$ and leading to terminal node 3 means that the element (1, 0, 3, 2) has three occurrences in the multiset *S*.

An MTMDD is denoted *ordered MTMDD* (OMTMDD) when a total order is defined on its variables (i.e., $x_l \succ x_k \Leftrightarrow l> k$) such that every path through the MTMDD visits nodes according to this ordering. It is important to notice that the choice of the ordering for the variables of an MTMDD can strongly affect the size of the MTMDD, i.e. its number of nodes. Unluckily, finding the optimal variable ordering is known to be a NP-complete problem [55]. As a consequence, the efficiency of application based on decision diagram data structures is strongly dependent on the development of domain-specific heuristics to select a good ordering. The MTMDD depicted in Figure 1(a) is hence an OMTMDD with the following variable ordering: $x_4 \succ x_3 \succ x_2 \succ x_1$.

Different reduction rules have been proposed to reduce significantly the number of nodes of the MTMDD without affecting the represented function. An OMTMDD is called reduced (ROMTMDD) if it contains neither redundant nor duplicated nodes. A redundant node is a non-terminal node *m* having all its children identical; i.e. $child(m, i) = child(m, j)$ for all $i, j \in \mathrm{I\!N}_{var(m)}$. As a consequence, the value of the function does not depend on the value of that variable. Duplicate nodes are two non-terminal nodes $m_1$ and $m_2$ labeled with the same variable and with identical children; i.e. $var(m_1) = var(m_2) \wedge child(m_1, i) = child(m_2, i)$ for all $i \in \mathrm{I\!N}_{var(m_1)}$.

In Fig. 1a the nodes *p* and *q*, colored in cyan, are an example of duplicate nodes, while *r* and *s* are an example of redundant ones. The corresponding ROMTDDD (i.e. without redundant and duplicated nodes) is instead reported in Fig. 1b. For sake of clarity, it is common to explicitly represent only those paths ending to the terminal nodes different from zero.

A common variation of the above reduction rule is to allow redundant nodes but no duplicate nodes. An OMTMDD is called quasi-reduced (QROMTMDD) if it contains no duplicate nodes and if all paths from the root node to a terminal node visit exactly one node for each variable. In Fig. 1c the quasi-reduced version of the OMTMDD in Fig. 1a is depicted.

Many DD packages implement the reductions stated above storing all the DDs in a single, multi-rooted graph structure, known as *unique-table* [56], where no two nodes are duplicated. In order to simplify, from this point on we shall refer to OMTMDD, ROMTMDD and QROMTMDD, simply as MTMDD.

MTMDDs can be manipulated applying the arithmetic operators (e.g. addition, multiplication, ...). Let $d_1$ and $d_2$ be two MTMDDs over the same domain, representing the functions $f_1$ and $f_2$, respectively, and let $\diamond$ be a generic binary operator. Then, the result of the $d_1 \diamond d_2$ operation is an MTMDD which encodes the function $f_1 \diamond f_2$. For instance, the multiplication between $d_1$ and $d_2$ results in an MTMDD such that the terminal node corresponding to the variable assignment $x_1, x_2, \dots , x_K$ is given by $f_1(x_1,\dots ,x_K)\cdot f_2(x_1,\dots ,x_K)$. The implementation of such operators is based on a recursive descent of the data structure and exploits a computed-table [56] to cache the result of each intermediate call to the algorithm. Figure 2 depicts the result of a multiplication between two MTMDDs. Only those variable assignments associated with value different from zero in both the factor MTMDD are reported, because the other assignments are linked to zero.

In the literature different software libraries implementing decision diagrams were proposed, such as CUDD [57], LibDDD [58] and Meddly [59]. In this work, we chose to use Meddly because of its efficiency and its ease of use. In fact, it provides a simple interface in which the complex aspects of using DDs (e.g. caching and garbage collection, ...) are automatically handled. Meddly, short for Multi-way and Edge-valued Decision Diagram LibrarY, is an open-source software library supporting natively MTMDDs, as well as a number of other types of DDs such as Binary Decision Diagrams, Matrix Diagrams and Edge-Valued MDDs. All DDs represented in Meddly are ordered and without duplicates. In Meddly, a named collection of decision diagrams associated with the same domain is called a *forest*. Within a forest, Meddly automatically removes duplicate nodes by means of a unique table, imposes reduction rules and handles memory management of the nodes.

Meddly provides two different user interfaces: a *basic interface* which provides the basic operators to easily create and manipulate DDs, and an *expert interface* which allows user to extend the existent operators and/or to integrate new ones. In this work, we implemented our tool by taking advantage of the basic interface of Meddly; in particular the following operators were exploited:

- createEdge() creates a new DD in the given forest by explicitly stating the return values for a set of variable assignments. Unspecified assignments are assumed to return a default value, which depends on the forest type (usually it is 0). For example, given the forest *F* and some variable assignments $Y=(y_1,\dots ,y_k)$, $W=(w_1,\dots ,w_k)$ and $Z=(z_1,\dots ,z_k)$, a call to F.createEdge(*Y*, *W*, *Z*, *a*, *b*, *c*) creates a new DD within *F* representing the function
  $$\begin{aligned} f(x_1,\dots ,x_k) = {\left\{ \begin{array}{ll} a &{} \text{ if } x_1=y_1 \wedge \dots \wedge x_k=y_k \\ b &{} \text{ if } x_1=w_1 \wedge \dots \wedge x_k=w_k \\ c &{} \text{ if } x_1=z_1 \wedge \dots \wedge x_k=z_k \\ 0 &{} \text{ otherwise } \end{array}\right. } \end{aligned}$$
- evaluate() determines the value of the function represented by the DD for a given assignment of its variables. Then, the call dd.evaluate($x_1,\dots ,x_k$) returns the terminal value linked to the path $x_1,\dots ,x_k$ of the decision diagram *dd*.
- apply() is used to manipulate DD applying on it a specific DD operator. Meddly supports both unary and binary operators and imposes that operands of binary operators must have the same domain, but they can live in different forests.

## Methods

GRAPES uses a trie, also known as prefix tree, to store the indexed graphs, since it provides a compact representation of a set of strings by taking advantage of their common prefixes, considerably reducing the data redundancy. In fact, a labeled path $(\sigma _1\sigma _2\dots \sigma _l)$ may be represented as a string $f_e(\sigma _1)f_e(\sigma _2)\dots f_e(\sigma _l)$ where $f_e:\Sigma \rightarrow {\mathbb {N}}$ is the mapping function from labels to the natural numbers.

Nevertheless, the tree structure of a trie (i.e. only a single edge can point to a node) makes it hard to exploit other types of symmetries present in the indexed graphs, as for instance the sharing of the same starting vertices and/or same relative occurrence number, as well as the sharing of common substrings which are not prefixes.

To deal with these aspects, in this work we propose to encode the indexed graphs into a DD, specifically an MTMDD: a trie generalization in which the requirement to have a tree structure is relaxed allowing multiple arcs to point to the same node. The main advantage of this is a potentially more compact representation due to the MTMDD ability to better exploit the regular structure of the data, such as common substrings present in the indexed graph paths. This allows the proposed methodology not only to reduce the memory utilization required to build and store the index, but also to reduce the time required for the pruning phase.

**Fig. 3:**
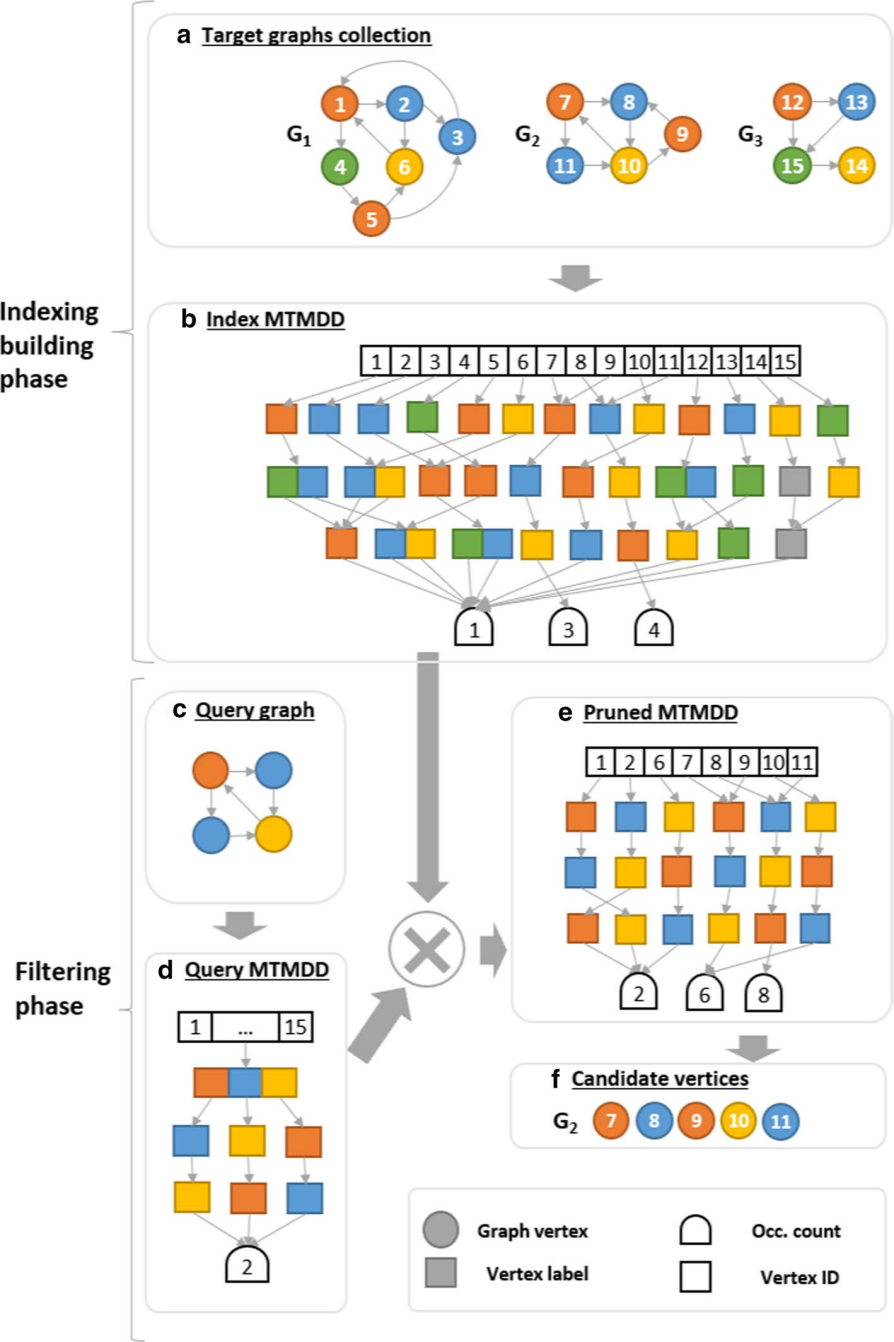
GRAPES-DD workflow with path length $l_p=3$

**Fig. 4:**
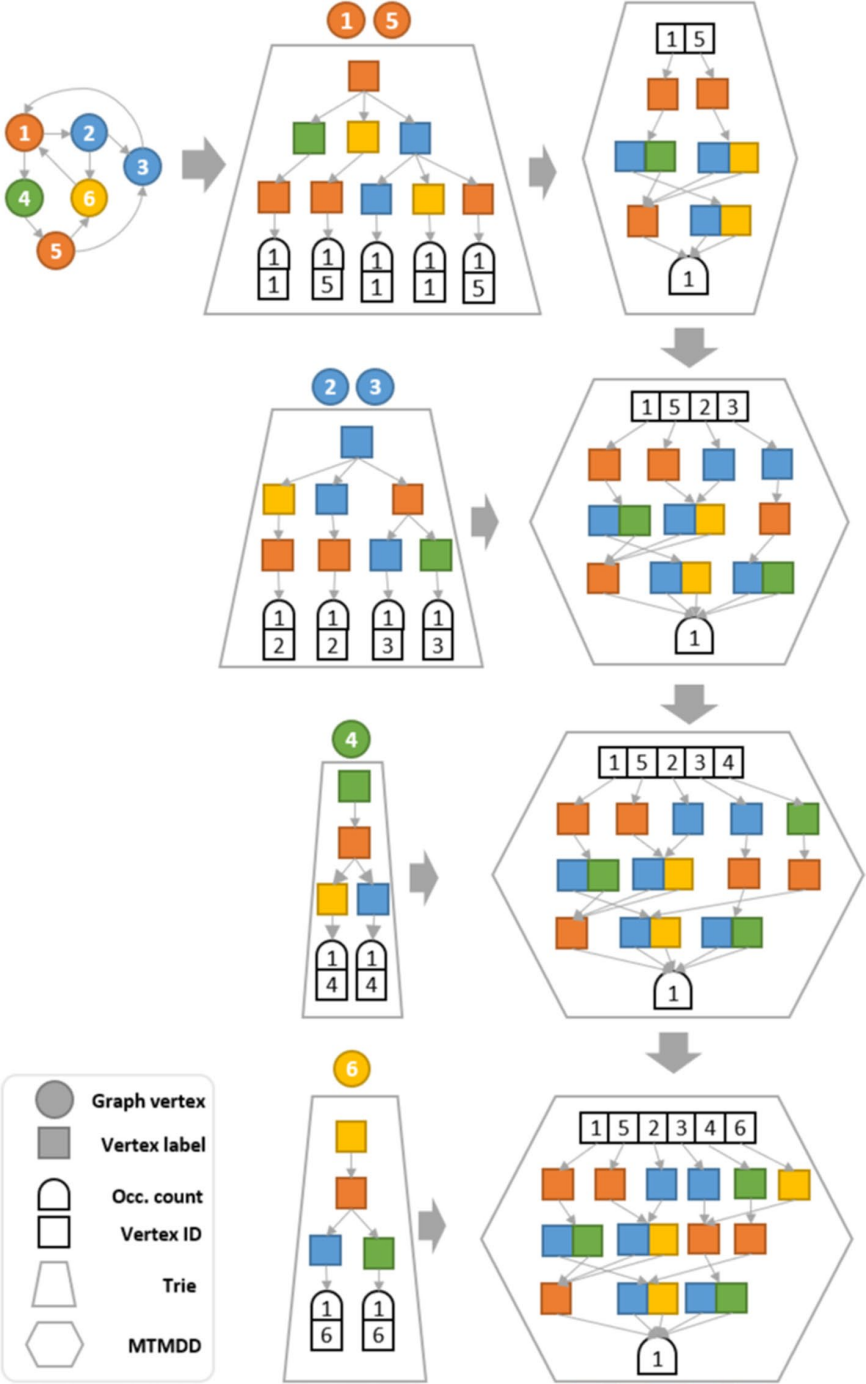
GRAPES-DD indexing of a target graph using a MTMDD built from partial tries

The GRAPES-DD workflow is reported in Fig. 3. The workflow is composed by two main phases: (1) the *index building phase* in which an MTMDD indexing the collection of target graphs is created, and (2) the *filtering phase* in which, given a query graph, the set of target graphs is potentially restricted to those sub-graphs probably containing the query. The GRAPES-DD verification phase remains as in the original version of the software (see Sect. 2.1.2 and [29] for details).

### Index building phase

The index building phase takes as input a collection of target graphs ${{\mathbb {G}}^t = \{ G^t_1, G^t_2, \dots , G^t_n\}}$ and the maximum path length $l_p$ and returns as output an MTMDD that maps each path to the total number of times it appears in the graph for a specific input vertex. We will refer to this MTMDD as *index MTMDD*.

In details, the first level of the index MTMDD stores the identification of the vertices of the indexed graphs. Then, the labelled paths are stored from the second to the last level of the MTMDD, one label per level starting from the first label of the path. Finally, the total occurrence number of the labelled paths in each indexed graph resides on the terminal nodes of the MTMDD.

An example of such a data structure is reported in Fig. 3b. This MTMDD is created considering the three target graphs, $G_1$, $G_2$ and $G_3$ in Fig. 3a and $l_p=3$. Since in the first level of the MTMDD all the vertices of the target graphs are enumerated, its domain is [1, 15]. Then, for each vertex in $G^t_i$ all the labelled paths up to length 3 starting from it are added in the MTMDD. Special nodes, namely *unlabelled* nodes and colored gray in Fig. 3b, are introduced to deal with labelled paths having length smaller than $l_p$. This is needed because MTMDDs cannot directly encode paths with different lengths.

Practically, the index MTMDD is created in an incremental manner, processing one graph at the time. Thus, the vertices of each graph are initially grouped based on their labels. For each of these groups, all the labelled paths containing up to $l_p$ vertices are retrieved by depth-limited search on the graph. These paths are stored temporally in a trie to efficiently count the occurrence of each labelled path in $G^t_i$. Before considering a new group, the trie is explored to create the corresponding MTMDD using the createEdge operator of Meddly and then is discarded. The created MTMDD is merged to the index MTMDD using the apply function with operator addition. Once the building phase is over, the MTMDD index is stored into a textual file.

Figure 4 shows the building steps for the target graph $G_1$ with $l_p=3$. The vertices of $G_1$ are indeed divided into four groups (i.e. $\{1,5\}$, $\{2,3\}$, $\{4\}$ and $\{6\}$) depending on their label. For each group the corresponding temporary trie and MTMDD are reported. The original GRAPES index is the union of the tries shown in Fig. 4. Thus, it contains the root node and 25 ($9+8+4+4$) nodes representing labelled paths, plus 13 nodes for storing the occurrences count, 13 nodes for storing the ids of the starting vertices in correspondence of each path, for a total of 51 nodes. On the contrary, the final MTMDD structure contains a single node storing all the six vertices ids, 13 nodes for the labelled paths and one node for the occurrence count, for a total of 15 nodes. In addition, the trie stores 39 links between its nodes, while the MTMDD stores 23 links.

### Filtering phase

The *filtering phase* takes as input the index MTMDD and a query graph $G^q$ that has to be searched within the graph collection. As output, the filtering phase provides for each graph the list of candidate vertices to match the query. Therefore, only these vertices will be subsequently tested using the subgraph isomorphism algorithm.

The algorithm initially builds the *query MTMDD* to represent the query graph through the use of its features (i.e. paths), which is shown in Fig. 3d. The vertices of the query graph are not represented in the corresponding MTMDD. The first level of such MTMDD contains all the vertex ids of $G^t_i$, meaning that initially each vertex of $G^t_i$ is candidate to match any path of $G^q$.

The multiplication operator (using the apply function) is applied between the index and the query MTMDDs, in order to extract from the index the information about the vertices really involved in the current query. We called *pruned MTMDD* the result of such multiplication, which is depicted in Fig. 3e. We see that only the subgraph composed by the vertices {1,2,6} is kept from $G_1$, $G_2$ is entirely kept and $G_3$ is totally discarded because it does not contain any feature in $G^q$.

The set of candidate vertices obtained is then filtered to keep only those graphs whose vertices satisfy the constraints imposed by the query. For each vertex $v_q$ of the query and a potentially matching vertex *v* of a target graph $G_i$, the algorithm verifies that (1) any path starting from $v_q$ also starts from *v* and that (2) the occurrence number of each path in the target graph is not less than the occurrence number of the same path in the query graph. Figure 3 shows that the graph $G_1$ is filtered out because the occurrence number of its features are not sufficient to satisfy the constraint imposed by $G^q$, while all the vertices of $G_2$ passed the filtering phase.

Finally, for each vertex of the query, the algorithm reports the list of the matchable vertices of the indexed graphs passing the pruning phase. The overall effect is that the algorithm extracts from the graph collection all the maximally connected components composed only by the vertices involved in the query graph. Over these components, the GRAPES subgraph isomorphism algorithm can be executed to find all the occurrences of the query graph [29].

## Results

### Datasets description

For this study, we considered six different types of graphs. Four of them are real graphs widely used as a benchmarks in the fields of bioinformatics and computational chemistry, the others are synthetically generated by means of the Barabasi–Albert’s and the Forest-Fire models. the choice of such two synthetic models has been taken according to their properties of the topologies to be similar the graphs used in biological databases. Differently from collections of real graphs, synthetic topologies allow us to investigate the performance of compared methods in relation to the parameters of such models, and thus to the properties of the produced topologies.

#### Biochemical structures

The collection of biochemical graphs was initially used for evaluating the performance of one-to-one subgraph isomorphism algorithms [60], and, nowadays, it is a well-established benchmark for graph theory problems linked to the subgraph isomorphism [61]. These four datasets that compose the collection are described in what follows.

*AIDS* is the standard database for Antiviral Screen [62], and it consists of 40k chemical structures representing small molecules. Vertices are atoms and edge are the chemical bounds linking them. Vertices are labelled by the atomic element they represent, and there are a total of 62 distinct elements. The average number of vertices per graph is 44.98, and the average degree is 4.17.

*PDBS* is a benchmark composed of 600 target graphs representing the topological structure of proteins [63, 64]. Vertices are the atoms and edges are chemio-physical bounds between them. These graphs have up to 16,431 vertices and 33,562 edges, with an average degree over the whole dataset equal to 4.27. There are a total of 10 unique labels, corresponding to the atomic types.

*PCM* is composed of three-dimensional structures of protein, called protein contact maps [65]. Vertices represent the amino acids of a protein and edges informs about the spatial proximity of amino acids. The dataset contains 200 target graphs having up to 883 vertices and 18,832 edges, with an average of 376 vertices per graph and 44.78 edges per vertex. There are a total of 21 labels of which 18 appears on average in each graph.

*PPI* is a dataset of 20 protein-protein interaction target graphs of 5 different species: *Caenorhabditis elegants, Drosophila melanogaster, Mus musculus, Saccaromyces cerevisae* and *Homo sapiens* [66]. Vertices are proteins and edges are predicted physical interactions between them. For each species, different thresholds on the accurateness of the prediction were applied, ranging from 0.4, 0.5, 0.6–0.7. Vertices are labelled according to their functional category, for a total of 45 distinct categories. The dataset contains graphs up to 10,186 vertices and 179,348 edges, an average degree of 18.46 and an average number of distinct labels per graph equal to 28.45.

For all of the biochemical datasets, queries were extracted from the target graphs by fixing the desired number of edges, from 4, 8, 18–32, and such that the topological structure of the extracted graph reflects the properties of the graph of origin.

#### Synthetic graphs

The Barabasi-Albert’s model is able to reproduce a graph with an observed stationary scale-free distribution, which reflects many of the structures that can be encountered in nature [67]. Starting from an initial set of vertices, $m_0$, the model inserts one vertex at time to the graph. At each insertion, new edges are added in order to connect the new vertex with existing ones. The probability of an edge with vertex *i* is $p_i = k_i^{\alpha }$, where *k* is the vertex degree and $\alpha$ is a user defined parameter. The benchmark contains 384 target graphs which were generated by fixing a desired number of vertices and average degree. Generated graphs have 200, 500, 1k, 5k, 10k and 20k vertices. In addition, three copies of each generated network are made in order to provide a labelled version of the initial structure with three different percentages of distinct labels, 0.1%, 1% and 10%. Labels are assigned randomly to vertices according to a uniform distribution.

The second type of synthetic graphs were generated according to the Forest-Fire model [68], that is inspired by forest growing behaviours, and which imposes a geometric distribution with mean $p/(1-p)$ which is used for randomly extract links between two distinct vertices. This benchmark contains 160 target graphs having the same number of vertices of the Barabasi-Albert benchmark, and they were labelled in the same way of the previous model. Moreover, the graphs were generated by varying the value of the model parameter *p* as 0.1, 0.3, 0.5, 0.7 and 0.9.

For both synthetic benchmarks, query graphs were extracted from the generated target graphs. The extraction was performed by fixing the number of desired vertices, ranging from 4, 8, 24, 32–64, and by extracting all edges among the selected vertices.

For more details regarding the two sets of synthetic benchmarks, the reader can refer to [69].

### Experimental setup and output

We evaluated the performance of GRAPES-DD, with respect to its predecessor GRAPES, by taking into account both space and time requirements. In particular, we focused on the amount of primary memory that the two approaches require during the execution, reported as *memory peak*, as well as the space needed to store the built index in the hard disk, reported as *index size*. In addition, we compared the running time required by the two approaches for building the index. The analysis was mainly focused on the index construction phase because it is the main difference between the two approaches. They share the same methodology for what concerns the matching phase. In addition, the filtering time can be considered negligible with respect to the total querying time.

Both GRAPES-DD and GRAPES have been containerized in a Docker [70] image in order to ensure both functional and computational reproducibility of the experiments. The dockerfile to build the image is provided together with the source code, and it is available at https://github.com/qBioTurin/grapes-dd or at https://github.com/InfOmics/grapes-dd. Both the tools were implemented in C++ and compiled with gcc 6.3.0. Then, the experiments have been carried out on a server equipped with four processors AMD Opteron 6167 2.20 GHz and 502 GB of RAM. Since GRAPES is a natively parallel software while GRAPES-DD is sequential, the experiments were executed using GRAPES with a single-thread.

**Fig. 5:**
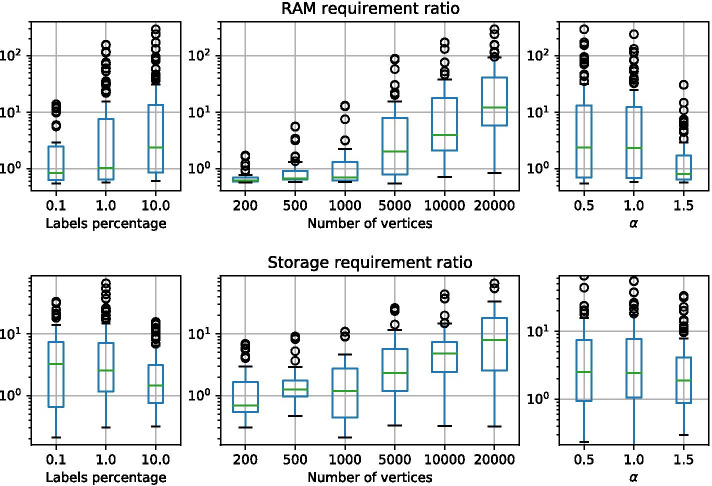
GRAPES/GRAPES-DD ratios of memory peak (as a RAM requirement) and index size (as a storage requirement), obtained by indexing Barabasi-Albert graphs. The chart was made by using the *boxplot* function of the Python3 Pandas module

**Fig. 6:**
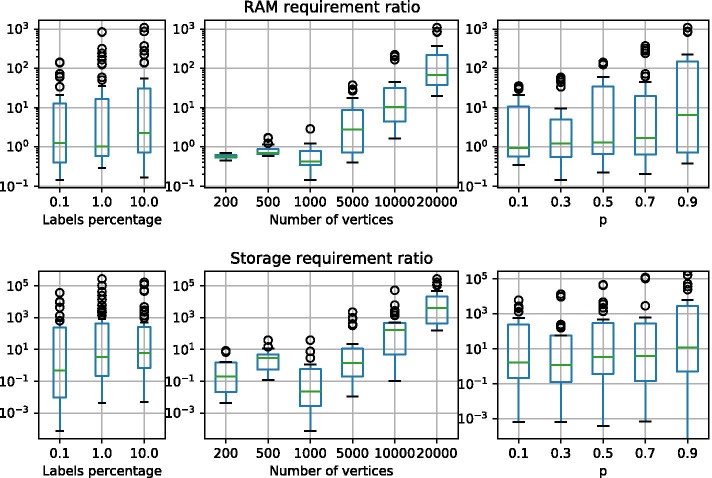
GRAPES/GRAPES-DD ratios of memory peak (as a RAM requirement) and index size (as a storage requirement), obtained by indexing Forest-Fire graphs. The chart was made by using the *boxplot* function of the Python3 Pandas module

Figures 5 and 6 show memory peak and index size on the synthetic datasets obtained by indexing one target graph at time. Values are calculated taking into account three different grouping strategies that reflect the way in which the datasets are generated. Plots were generated via the *Pandas* framework available for Python^3^. In details, datasets were grouped by (1) percentage of distinct labels with respect to the total number of vertices of the graph, (2) number of vertices and (3) value of the Barabasi-Albert model parameter $\alpha$ or Forest-Fire parameter *p*.

Results show that, independently from the label percentage and model parameters, the performance of GRAPES-DD improves as the number of vertices of the indexed graph increases. In fact, for graphs having less than 5k vertices, the memory peak required by GRAPES-DD is higher than the peak of GRAPES, resulting in a ratio between the two values less than 1. The out-performance of GRAPES-DD can reach two/three orders of magnitude with respect to GRAPES, for graphs with 20k vertices, which means that the memory requirement of GRAPES-DD is one hundredth that of GRAPES.

Similar trends are observed for the size of the index when it is stored into the hard disk. In this case, the ratio can achieve five orders of magnitude as it is shown for the Forest-Fire graphs with 20k vertices. In general, best ratios are obtained for the Forest-Fire graphs with a high number of vertices, however, this behaviour is counterbalanced by the fact that on average Forest-Fire graphs with less than 5k vertices are also those with the lowest ratios.

For what concerns the memory peak, we can observe that the label percentage is a more crucial factor for the Barabasi–Albert model rather than for the Forest-fire model. More in general, a low label percentage is to the advantage to the trie structure of GRAPES because the extracted paths share and relatively high number of labels. Opposite trends are observed for what concerns the storing of the index.

As for the label percentage, model parameters produce less variation compared to the number of vertices. The Barabasi-Albert model produces scale-free networks where the distribution of the degrees of the vertices follows a power law. A value greater than 1 increases the skewness of the resultant distribution, while a value less than 1 flattens the distribution. Thus higher values trend to produce a more sparse graph. Results in Fig. 5 show that GRAPES-DD performs better for dense graphs, namely for low values of the $\alpha$ parameter. The trend is confirmed by the results regarding the Forest-fire models (Fig. 6), where higher values of the *p* parameters produce more dense graphs.

GRAPES-DD reaches an average indexing compression ratio of 11.16 with respect to GRAPES when Barabasi-Albert networks are indexed. Instead, an average ratio of 9.46 is reached over the Forest-Fire collection. The better ratio obtained by GRAPES-DD highlights that the application of MTMDDs is advantageous for any of the two types of random graphs, however, it is more suitable for Barabasi-Albert networks that are considered more similar to biological networks.

**Table 1:** Indexing comparison of GRAPES and GRAPES-DD of synthetic graphs in terms of RAM requirement, Storage requirement, and Building time

			RAM req. (MB)			Storage req. (MB)			Build time (s)	
			GRAPES-DD	GRAPES	ratio	GRAPES-DD	GRAPES	ratio	GRAPES-DD	GRAPES
Barabasi-A.	*l*	0.1%	3649	7935	2.2	305	3,493	11.5	470	109
		1%	8229	66,838	8.1	1543	28,772	18.6	646	214
		10%	7876	81,552	10.4	10,071	34,368	3.4	668	265
	$\alpha$	0.5	2103	94,654	45.0	24,915	40,281	1.6	516	330
		1	8351	58,519	7.0	16,602	25,145	1.5	766	213
		1.5	1447	3068	2.1	1246	1219	1.0	144	26
Forest-Fire	*l*	0.1%	834	3,929	4.7	121	1,689	13.9	63	29
		1%	1308	21,255	16.2	738	9,229	12.5	77	66
		10%	1167	24,936	21.4	5,351	10,650	2.0	62	70
	*p*	0.1	147	1426	9.7	2,882	585	0.2	10	7
		0.3	188	2451	13.1	3,358	1,024	0.3	14	9
		0.5	281	4966	17.7	3,922	2,109	0.5	26	16
		0.7	487	11,694	24.0	4,535	5,020	1.1	57	37
		0.9	988	29,565	29.9	5,386	12,840	2.4	139	88

Subsequently, we evaluated the performance of exploiting the MTMDD structure for indexing 14 collections of synthetic graphs (see Table 1). The first three collections are obtained by grouping Barabasi–Albert graphs by the label percentage, such that graphs having the same percentage are contained in the same collection. Similarly, Forest-Fire graphs were grouped into three further collections. The grouping procedure was also performed by taking into account the $\alpha$ and *p* model parameters. As for the previous analysis, the ratio is computed by dividing the values measured for the trie structure of GRAPES with those registered for the MTMDD of GRAPES-DD. As it has been shown for the single-graph analysis, the percentage of distinct labels with respect to the total number of vertices in the graphs) is a discriminant factor for the compression gain obtained by the MTMDD structure. Also the trends relative to the parameters of the models are confirmed. In general, the MTMDD structure is on average more convenient on the Forest-Fire graphs for what concerns the memory peak. Barabasi-Albert graphs with $\alpha = 0.5$ are an exception to this trend, since they reach the maximum registered ratio equal to 45. In contrast to the single-graph analysis, the space required for storing the index into the hard disk does not provide the same advantage to the MTMDD structure. In fact, in the single-graph analysis the ratio reaches a value of $10^5$ that is two order of magnitude higher of the ratios obtained for the memory peak. On the contrary, these experiments show an inversion of the ratio such that the MTMDD structure reaches best results for the memory peak. It is notable to report that, while the trie structure requires a maximum of 94Gb of memory, the process for building the MTMDD-based index does not reaches the 9Gb of requirement, making it suitable for common personal computers.

Table 1 also shows the running time of the two approaches for building the index and for storing it. The MTMDD structure requires more time for its construction, the compression capability of the MTMDD must come with an unavoidable additional cost. However, the growth time is only a few minutes and the construction of the index is performed in a preprocessing phase, only once and reused for each query search.

Table 2 reports the complete set of experiments that were performed on the biochemical graphs. The experiments regard the indexing of the four different collections of real graphs. For this benchmark, ratios are less prominent compared to synthetic graphs, however many of them are higher than 1, confirming a gain in using the MTMDD structure rather than the trie. The trend for which paths extracted from more dense and more uniform graphs are better compacted by the MTMDD structure is confirmed. In fact, the best ratio is obtained for the *PCM* collection that contains the most dense graphs. However, the *PCM* collection is also the one with the lowest number of labels and a relatively small number of vertices. Thus, it seems that the density of the graphs is the key factor for the good performance of GRAPES-DD in biochemical graphs. In addition, in contrast with the results on the synthetic graphs, the running time of GRAPES-DD for the construction of index is generally faster than the time required by GRAPES. In these cases, the compression capability of the MTMDD comes without additional cost.

**Table 2:** Indexing comparison of GRAPES and GRAPES-DD of biochemical datasets in terms of RAM requirement, Storage requirement, and Building time

	RAM req. (MB)			Storage req. (MB)			Build time (s)	
	GRAPES-DD	GRAPES	ratio	GRAPES-DD	GRAPES	ratio	GRAPES-DD	GRAPES
*AIDS*	5304	1064	0.20	164	39	0.24	170.12	16
*PDBS*	532	556	1.04	22	17	0.78	176.00	10.07
*PCM*	512	7057	13.77	253	1,392	5.51	617.24	754.56
*PPI*	629	1698	2.70	166	665	4.00	2514.18	2906.65

**Table 3:** Indexing comparison of GRAPES and GRAPES-DD of single PPI network in terms of RAM requirement, Storage requirement, and Building time

			RAM req. (MB)			Storage req. (MB)			Build time (s)	
Species	|*V*|	|*E*|	GRAPES-DD	GRAPES	ratio	GRAPES-DD	GRAPES	ratio	GRAPES-DD	GRAPES
*S. cerevisiae*	4709	40,284	38.1	91.1	2.39	10.5	26.2	2.49	60.93	63.49
	5,230	53,699	56.3	136.3	2.42	11.7	42.4	3.63	604.47	630.92
	5,762	76,482	61.1	150.9	2.47	12.7	47.5	3.73	846.24	879.90
	5,936	89,674	46.5	121.2	2.61	12.9	36.8	2.86	128.52	135.01
*C. elegans*	1557	2472	7.1	5.7	0.80	0.2	0.3	1.45	0.24	0.20
	2421	3981	7.8	7.9	1.02	0.5	1.0	2.06	0.52	0.44
	3664	7005	10.4	14.6	1.40	1.0	2.8	2.65	1.33	1.13
	6173	26,184	25.1	58.5	2.33	4.3	16.0	3.75	34.16	34.19
*D. melanogaster*	1185	2008	8.0	12.1	1.51	0.6	1.5	2.30	0.31	0.26
	2488	6151	12.1	32.1	2.65	1.9	6.6	3.47	3.28	3.21
	2729	7235	13.3	36.2	2.73	2.3	7.9	3.37	4.30	4.22
	7928	37,542	52.3	198.7	3.80	13.1	64.3	4.90	144.05	156.07
M. musculus	1810	2413	8.0	13.1	1.64	0.7	2.2	2.94	0.42	0.36
	3255	5424	11.0	31.0	2.81	1.9	7.1	3.68	2.52	2.50
	3758	6853	13.1	43.6	3.33	2.6	11.2	4.30	4.47	4.61
	6875	23,779	41.1	193.6	4.71	12.4	62.1	5.02	76.64	81.56
*H. sapiens*	4638	10,665	17.3	55.0	3.18	3.6	14.6	4.07	5.60	5.36
	8728	31,164	53.5	215.4	4.02	13.1	70.8	5.42	65.14	68.26
	9826	48,835	87.5	351.4	4.02	21.5	120.1	5.59	213.95	230.16
	10,186	51,484	89.2	391.8	4.39	22.0	134.1	6.10	191.63	209.63

Collections of biochemical graphs were also used for evaluating the performance of GRAPES-DD during the querying phase in comparison with exiting approaches VF2 [21] and CT-Index [31]. VF2 is a non-indexed approach used by GRAPES and GRAPES-DD in the verification phase. The comparison with it allows us to evaluate the effectiveness of using indexing in graph searching applications. CT-Index is a index-based graph searching solution that uses paths as indexing features. Biochemical graphs have already been used for investigating the performance of GRAPES, VF2 and CT-Index [29, 48]. Here, we propose those comparisons by adding GRAPES-DD. GRAPES-DD is compared with GRAPES, two configurations of CT-Index and the pure subgraph isomorphism algorithm VF2. All the compared methods enumerate all the matches. CT-Index was run with default parameters (CT-index def), such that paths, cycles and trees are indexed. Moreover, a configuration (*CT-index 4*) which only includes paths of length 4 was taken into account. We were not able to run CT-Index on the PCM and PPI datasets due to excessive memory usage of the tool.

**Fig. 7:**
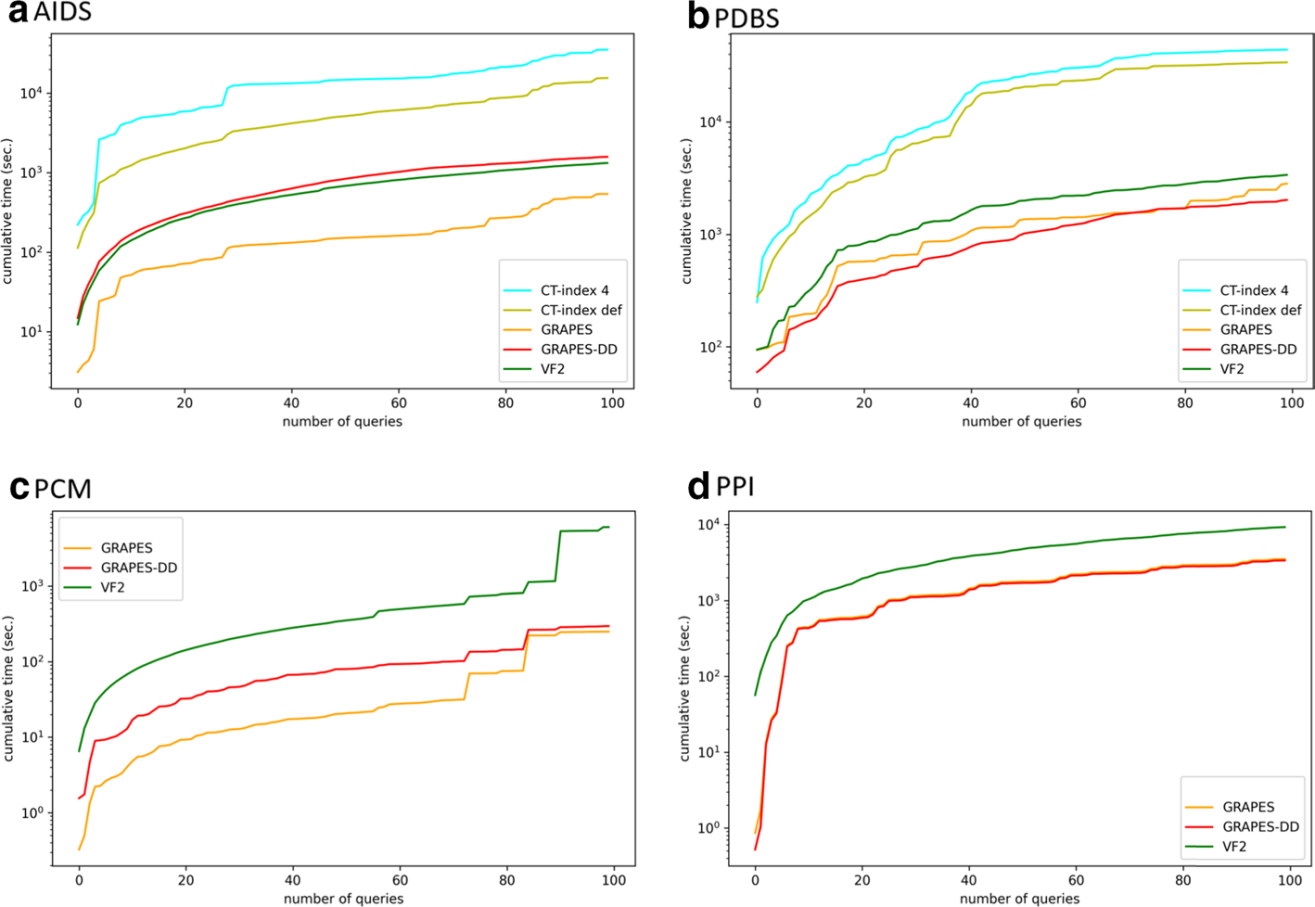
Cumulative time for running 100 queries over the four collections of biochemical graphs. The chart was made by using the *plot* function of the Python3 Pandas module

Figure 7 shows the cumulative time (in seconds) for executing 100 queries. Running times of GRAPES, GRAPES-DD and CT-index include the time to read graphs from the input files, filtering time and verification time. The time required by the methods for reading the pre-built index is considered only once and it is included in the running time of the first executed query. Since no index is built by VF2, its total execution time is taken into account. CT-index takes 461 seconds for building the index of the AIDS datasets with default parameters and 82 seconds for indexing only paths of length 4. Moreover, it requires 4,400 for indexing the PDBS collection with default parameters and 40 seconds when only paths of length 4 are taken into account. In all experiments, CT-index is outperformed by the other three approaches.

On AIDS collection (see Fig. 7a), GRAPES-DD is not able to outperform GRAPES; its running time is close to the one of VF2. As shown in Table 2, this type of biochemical structures are too small and not suitable for being indexed and queried via MTMDDs. The overhead for reading the index and for extracting candidate graphs according to the query structure is not amortized during the verification phase, indeed, GRAPES-DD requires 15 seconds for reading the index and an average of 12 seconds for the filtering phase. On the contrary, GRAPES requires only 3.5 seconds for loading the index and an average of 3 seconds for the filtering.

The VF2 algorithm is outperformed by GRAPES-DD in the PDBS, PCM and PPI collections (see Fig. 7b–d). Moreover, VF2 is outperformed by GRAPES also in AIDS dataset. Thus, the index-based methodology used by GRAPES and GRAPES-DD is generally helpful in reducing the time required for the verification phase.

Regarding the PDBS collection (see Fig. 7b), GRAPES-DD requires 2.3 seconds for loading the index and an average of 6 seconds for filtering the collection. GRAPES requires 0.12 seconds for the index load and 20 seconds for the filtering phase. Since GRAPES-DD and GRAPES produce the same set of candidate graphs, GRAPES-DD outperforms GRAPES thank to its performance in the filtering phase.

Considering the PCM collection (see Fig. 7c), GRAPES-DD requires 20 seconds for loading the index and an average of 14 seconds for filtering the collection. GRAPES requires 30 seconds for the load and 2 seconds for the filtering. Thus, GRAPES-DD builds a more succinct index that allows a fast loading time, however it is not sufficient for outperforming GRAPES in filtering time.

On the PPI collection (see Fig. 7d), GRAPES-DD and GRAPES have comparable running times. GRAPES-DD requires 13 seconds to load the index, in contrast to 2 seconds required by GRAPES. However, GRAPES-DD spends on average 0.05 seconds for the filtering phase, while GRAPES requires on average 11 seconds.

Lastly, Table 3 reports the results regarding the PPI networks obtained by indexing one PPI at time, since PPI networks are often analysed stand-alone. Similarly to the synthetic networks, the increase of the graph size, i.e. number of vertices |*V*| and number of edges |*E*|, results in a better performance of GRAPES-DD with respect to GRAPES. However, comparing the ratios obtained for *M. musculus* and *H. sapiens* we can deduce that as expected there is not a fixed correlation between the graph size and the performance. Therefore, the intrinsic nature of the graph is also responsible for these results. PPI networks are also the targets for which running times of GRAPES-DD are comparable to those of GRAPES, and some times they are even better. The GRAPES-DD building approach includes the construction of partial tries but without merging them. The cost for traversing a single whole trie may limit GRAPES.

## Discussion

In this study, we deal with the problem of reducing the indexing size of biochemical and biological graph searching systems to make them effective with the increasing size of the structures. We show that the indexing of labelled graphs can take the advantages of newly adapted data structures based on decision diagrams. These techniques allow already existing methodologies to increase their compression power, in terms of memory consumption, without significantly increasing the searching time requirement.

We examined synthetic graphs because they offer a more systematic way of investigating performance of indexing using decision diagrams. Since the type of the generated graphs reflects the structures that are found in nature, their analysis can be exploited for inferring performance behaviour of real biochemical and biological structures. The results showed that relevant indexing compression ratio can be obtained in relation with the size and the topological structure of the graphs and the distribution of labels within them. Moreover, the larger are the indexed graphs, the higher is the advantage of using Decision Diagram data structure.

A well-established benchmark was also used for evaluating the performance on real graphs. The size of the considered graphs are relatively small, compared with the synthetically generated ones, however trends of gain ratio are confirmed. This must be considered in the perspective of future applications of the proposed indexing technique, because the continuous development of new technologies for extraction biological information leads to the construction of biological relational systems that constantly increase in size. In addition to the gain in compression ratio, GRAPES-DD outperforms GRAPES in terms of build times while maintaining comparable query times. Furthermore, our analyse show that graph search approaches based on indexing, in graphs of some complexity, can amortize the overhead of building indexing data structures at query time.

## Conclusions

Nowadays, graphs are fundamental structures for representing and for investigating the current biological and biomedical knowledge. In this work we investigated the possibility to improve the performance of the cutting-edge algorithms for searching substructures in graphs based on indexing, by addressing one of their disadvantages which is the size of the index. To this aim, we developed GRAPES-DD, a new version of GRAPES tool, whose strength is the use of decision diagrams to substantially reduce the size of the index. Experimental results performed on a set of synthetic and real benchmarks reported clearly that the use of this data structure allows us to substantially reduce the memory footprint of the index (i.e up to 5 orders of magnitude smaller) with respect to the original version of GRAPES without impacting the running time of the algorithm.

Further enhancement of GRAPES-DD will be to re-implement the building phase to allow thread-based parallelization as in the original GRAPES implementation. Moreover, since the efficacy of decision diagram techniques is strictly dependent on the variable order, we will investigate how different algorithms for variable orderings behave and we will evaluate the possibility of developing meta-heuristics to identify a-priori the best variable ordering depending on the features of each target graph.

## Data Availability

Project name: GRAPES-DD Project home page: https://github.com/InfOmics/grapes-dd and https://github.com/qBioTurin/grapes-dd Source code repositories: https://github.com/qBioTurin/grapes-dd and https://github.com/InfOmics/grapes-dd Operating system(s): Linux Programming language: C++, Docker License: MIT Any restrictions to use by non-academics: licence needed

## Abbreviations

DD: Decision diagram
MTMDD: Multi-terminal multi-way decision diagram
OMTMDD: Ordered multi-terminal multi-way decision diagram
ROMTMDD: Reduced ordered multi-terminal multi-way decision diagram
QROMTMDD: Quasi-reduced ordered multi-terminal multi-way decision diagram
SMP: Symmetric MultiProcessing
PPI: Protein–protein interaction

## Footnotes

1 https://het.io/.

2 The multiset (or bag) extends the concept of a set allowing for multiple instances for each of its elements.

3 https://pandas.pydata.org/pandas-docs/stable/reference/api/pandas.DataFrame.boxplot.html
